# Colour Variation in the Crocodile Lizard (*Shinisaurus crocodilurus*) and Its Relationship to Individual Quality

**DOI:** 10.3390/biology11091314

**Published:** 2022-09-04

**Authors:** Xia Qiu, Martin J. Whiting, Weiguo Du, Zhengjun Wu, Shuyi Luo, Bisong Yue, Jinzhong Fu, Yin Qi

**Affiliations:** 1 Chengdu Institute of Biology, Chinese Academy of Sciences, Chengdu 610041, China; 2 College of Life Sciences, Sichuan University, Chengdu 610065, China; 3 University of Chinese Academy of Sciences, Beijing 100049, China; 4 School of Natural Sciences, Macquarie University, Sydney, NSW 2109, Australia; 5 Institute of Zoology, Chinese Academy of Sciences, Beijing 100101, China; 6 Key Laboratory of Ecology of Rare and Endangered Species and Environmental Protection, Guangxi Normal University, Ministry of Education, Guilin 541001, China; 7 Guangxi Key Laboratory of Rare and Endangered Animal Ecology, Guilin 541001, China; 8 Daguishan National Nature Reserve for Crocodile Lizards, Hezhou 542800, China; 9 Departments of Integrative Biology, University of Guelph, Guelph, ON N1G 2W1, Canada; 10 Mangkang Biodiversity and Ecological Station, Tibet Ecological Safety Monitor Network, Changdu 854500, China

**Keywords:** colour signal, colour polymorphism, social communication, alternative reproductive tactic, reptile, life history

## Abstract

**Simple Summary:**

This study examines colour variation in the highly endangered crocodile lizard, *Shinisaurus crocodilurus*. Both males and females vary in the extent to which their throats and venters are red. Their colouration is easily visible to a lizard receiver, and we found evidence that colour signals individual quality. Females with red venters had larger heads while females with red throats had greater bite force. In males, redder individuals were older. Finally, we found links between colour and fitness in males but not females. Aspects of male colouration were linked to reproductive output such that they sired offspring from heavier litters. The potential fitness consequences of colour should be considered in captive breeding and release programs.

**Abstract:**

Colour plays a key role in animal social communication including as an indicator of individual quality. Using spectrophotometry, we examined colour variation in the throat and venter of the crocodile lizard (*Shinisaurus crocodilurus*), an endangered species native to southern China and northern Vietnam. We detected two broad colour variants, individuals with and without red, for each body region and each sex. A cluster analysis of spectral colour measurements (hue, chroma, luminance) revealed discrete throat and ventral morphs when measured in a single snapshot in time. However, photographic evidence revealed that the amount of red relative to body size increased as they got older. Individuals with red were equally likely to be male or female and throat colour was unrelated to ventral colour. Therefore, it is premature to claim that crocodile lizards have discrete colour morphs. We used visual modelling to show that the throat and venter were easily discriminable to a lizard visual system, suggesting they function in social communication. We also asked whether colour variation signalled individual quality. Females with red throats had greater bite force while males with red throats were older. In addition, females with red venters had larger heads. We also detected differences in morphology linked to colour. Females with red throats had slender bodies and longer tails, while individuals lacking red on their throats were stouter and had shorter tails. Finally, throat and ventral colour were unrelated to reproductive output (litter size and mass) in females. Males with greater ventral luminance contrast sired offspring from litters with greater litter mass (including stillborns), while males with greater ventral chromatic contrast sired offspring whose collective live mass (excluding stillborns) was greater. Males with greater luminance contrast also sired more live offspring (excluding stillborns). Collectively, these results suggest that male ventral colour signals individual quality in males. Conservation initiatives should take colour variation into account when planning future captive breeding and release programs for this endangered species.

## 1. Introduction

Animal colour signals directly bear on fitness and consequently, their evolution and function has attracted considerable attention from evolutionary biologists and behavioural ecologists [1,2]. Selections typically act on colour signals to increase their conspicuous to a conspecific against the signalling environment (e.g., increasing contrast to the habitat background) [3]. In addition, colour conspicuousness is also largely dependent on the receiver’s visual system, which determines spectral sensitivity and colour perception [1,4]. Due to the complex nature of social communication among individuals of varying age and social status, combined with selections imposed by the environment on signal structure and conspicuousness, colour signals show a wide range of variation [2,5,6].

One important role of colour variation is to facilitate individual and social recognition, which may involve three processes [5]. First, colour variation may signal individual identity in social contexts where individuals repeatedly interact with each other, such as facial markings in wasps [7]. Second, colour variation may convey information about the quality of the signaller, thereby improving assessment between rivals and reducing conflict. For example, the redness of abdominal ornamentation in male European minnows (*Phoxinus phoxinus*) signals body condition and swimming performance [8], while aspects of ultraviolet colouration signal fighting ability in Augrabies flat lizards (*Platysaurus broadleyi*) [9]. Third, colour variation may co-evolve with morphology, behaviour and life history. For example, throat colour variation in side-blotched lizards (*Uta stansburiana*) represents discrete alternate reproductive strategies that correlate with specific behavioural and life history traits [10]. Nevertheless, the majority of studies of colour variation have focused on males, while research on females is limited [2,11,12,13].

**Figure 1:**
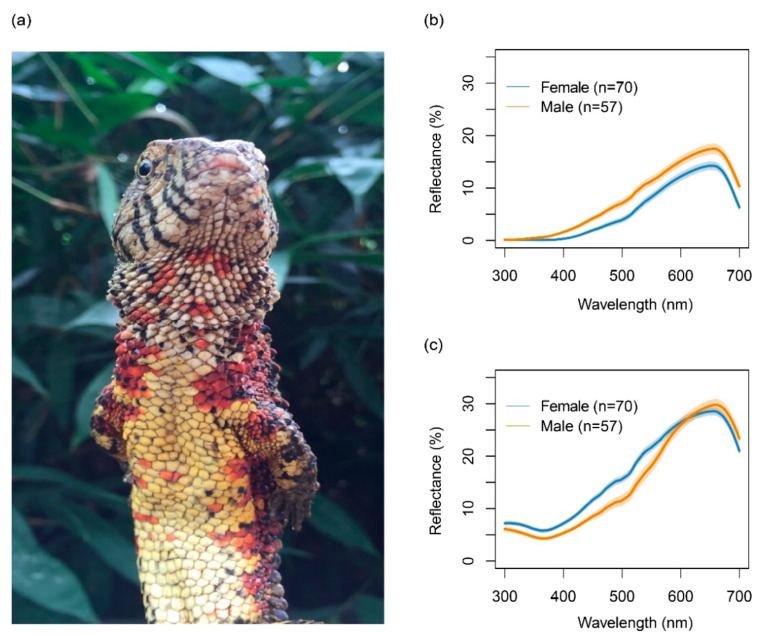
(**a**) A male crocodile lizard (*Shinisaurus crocodilurus*) with red colour patches on the throat and venter. (**b**) Spectral reflectance curve for the throat by sex. (**c**) Spectral reflectance curve for the venter by sex.

The crocodile lizard (*Shinisaurus crocodilurus*) has extensive colour variation in both males and females, particularly in the throat and ventral regions (Figure 1a). He et al. [14] reported sexual dimorphism with males being light blue or bright red and females as light yellow or red. Van Schingen et al. [15] reported blue, red and yellow throat morphs. Nevertheless, previous research on colour variation in *S*. *crocodilurus* is largely subjective. *Shinisaurus* *crocodilurus* has been listed as an endangered species since 2014 [16] and has been listed in CITES Appendix I due to the threats from habitat degradation and the international pet trade. Its current natural distribution includes south-eastern China and north-eastern Vietnam [17]. A survey of the Chinese population in 2004 estimated 950 individuals in the wild [18], while the Vietnamese population was estimated to be less than 200 individuals in 2016 [15]. Therefore, *S. crocodilurus* is critically endangered. Assessing the links between colour variation and individual quality may facilitate captive breeding and release programs for this species. In addition, the potential links between colour variation and individual identity may be useful in individual recognition.

Here, we examined colour variation in *S*. *crocodilurus*, particularly in the throat and ventral regions, using spectrophotometry. Our objectives were to (1) quantify colour variation and establish whether lizards of both sexes can be assigned to discrete colour morphs; (2) use visual modelling to quantify the conspicuous of colour patches to conspecific receivers; and (3) examine the relationship between colour and indices of individual quality (e.g., body condition, bite force) and reproductive output (litter size, litter mass).

## 2. Materials and Methods

### 2.1. Study Site

All lizards in this study were part of a captive breeding population in the Guangxi Daguishan Chinese Crocodile Lizard Nature Reserve (24°09′ N, 111°81′ E) in China. The captive breeding centre is located inside the reserve where the lizards naturally occur and consists of semi-natural outdoor enclosures (L: 3 m × W: 2 m × H: 1.1 m). To simulate their natural habitat, water is pumped from nearby streams and flow through each breeding enclosure, which also contain rocks and bushes (*Saurauia tristyla*) to give lizards adequate cover. These lizards experience the same climatic factors and similar habitats as their wild counterparts.

### 2.2. Colour Measurements of Lizards and Their Habitat

We measured the colouration of 127 adult lizards (70 females, 57 males) in May–June 2017. Specifically, we measured the conspicuous orange/red colour patches visible to the naked eye on the throat and venter, using a Jaz optic spectrophotometer (Ocean Optics Inc., Dunedin, FL, USA) with a PX2 light source. For each individual, we measured three separate locations on the throat and nine on the venter (Figure 1), even when there was no obvious colour patch (i.e., no red). All measurements were taken relative to a dark and white 99% WS-1 (Labsphere) standard, using a fibre-optic reflectance probe placed at 90° to the skin surface at a fixed distance of 5 mm. For each individual, we also took a photo of the centre and the side of the body. The sex was determined by checking for hemipenal bulges. We also obtained photos of the entire ventral surface of 32 lizards from successive years from birth to adulthood, which allowed us to examine the ontogenetic development of colour on the throat and venter. Individuals at least two years old are regarded as adults.

In order to quantify their signalling environment, we measured the spectral reflectance of green leaves on trees (n = 57) from their habitat. Additionally, we measured absolute irradiance (n = 47) using a separate irradiance channel held at ground level and parallel to the ground, thereby capturing side-welling light. All measurements were taken during the peak period of the lizard’s activity (10:00–12:00 h) and used in visual modelling of conspicuousness.

### 2.3. Colour Variation

We explored the possibility of colour-specific morphs by performing a spectral analysis of hue, chroma and luminance for the throat and venter of males and females. We used the “*kmeans*” function from *stats* package in R [19] to test whether colour patches from different body regions and sexes formed distinctive colour variants. This analysis does not take the animal’s visual system into account but can be informative as cluster analysis. We calculated hue, chroma and luminance from 127 individuals for the cluster analysis. Colour measurements from multiple patches of the same region were averaged for each individual. First, we tested for the best *k* value using the sum of squares error (SSE) optimized by using the elbow method [20]. The lowest value of SSE and the elbow of the curve represent the optimal *k* value (Figure S1). We then assigned the colour (averaged from multiple patches) of each body region into a type based on the optimal number of clusters (*k*).

We further examined the ontogeny of colour development in 32 lizards (21 females, 11 males) using photographs across multiple years to establish whether they corresponded to discrete colour morphs (i.e., polymorphism sensu [2]). These photographs included their first year and some or all subsequent years into their adulthood. Discrete morphs should be relatively invariant with respect to colour once they reached adulthood.

### 2.4. Conspicuousness of Colour to the Lizard Visual System

We used the receptor noise limited (RNL) model in the R package *pavo* [21,22] to calculate chromatic (∆S) and luminance (∆L) contrast for the throat and venter regions against background vegetation. Prior to analysis, we reduced each spectrum to between 300 and 700 nm, smoothed each individual’s spectral reflectance curve to remove electrical noise, and corrected negative values. We also averaged the spectral reflectance of each body region. The spectral sensitivity data for *S*. *crocodilurus* were not available, so we used those of another diurnal lizard, the agamid *Ctenophorus ornatus* [23] assuming that lizard visual systems are conservative [24]. We assumed λmax for the UVS, SWS, MWS and LWS to be 360 nm, 440 nm, 493 nm and 571 nm, respectively, along with photoreceptor class densities of 1:1:3.5:6 (UVS:SWS:MWS:LWS). We used a signal-to-noise ratio (weber fraction) of 0.10 in all cases. We calculated discrimination thresholds as just noticeable differences (JNDs) for each colour patch. JND values greater than one are traditionally assumed as the threshold of discrimination between two colours, and JNDs greater than three indicate that two colours can be discriminated easily [25]. To reflect the sexual differences in conspicuousness, we calculated the JNDs for males and females separately. We also calculated the chromatic and luminance contrast of each colour variant for the throat and venter separately.

We tested for differences in conspicuousness (JNDs) between body regions, sex and the interaction between them using linear mixed models (LMM) in the R package *lme4* [26]. We also tested for differences in conspicuousness between colour variants for throat and venter separately. JNDs were log-transformed and treated as dependent variables, with body regions, sex, interaction between body regions and sex as independent variables, while assuming a Gaussian error distribution. A family group parameter, which describes the full sibling relationship between individuals, was used as a random effect to satisfy independence assumptions. Residuals were tested by using the “*qqnorm*” function in the R *stats* package [19] to satisfy the assumption of normality.

### 2.5. Morphology and Bite Force Measurements

We measured body mass to the nearest 0.1 g on a digital scale, snout–vent length (SVL) and tail length to the nearest 1 mm using a plastic ruler, and head length, head width and head height to the nearest 0.01 mm using digital calipers. We used a principal components analysis to summarize three head measurements into a single measure, PC1, which explained 86.4% of the total variation in head size. We also calculated the scaled mass index (SMI), which is widely used as an indicator of individual body condition [27]. It also provides information on body shape (slender vs. stout). SMI was computed as follows:

$$SMI=M_{i}{\left[\frac{L_{0}}{L_{i}}\right]}^{b_{SMA}}$$

where *M_i_* and *L_i_* are the body mass and SVL of individual *i*, respectively, *b_SMA_* is the scaling component estimated by SMA regression of M on L, and *L*_0_ is mean value for the SVL.

We measured bite force using a piezoelectric force transducer (Type 9203, Kistler Inc., Winterthur, Switzerland), connected to a charge amplifier (Type 5995A, Kistler Inc., Winterthur, Switzerland), and fitted with two plastic plates following Vanhooydonck et al. [28]. We encouraged lizards to bite the plates by tapping them softly on the side of the mouth. Bite force was measured three times, and the maximum value was used in the analyses [28]. To account for the potential variation in bite force due to individual body temperature, we measured the lizard’s body temperature using an infrared thermometer (HCJYET) before measuring bite force.

### 2.6. Age, Parentage and Reproductive Output

The captive breeding centre uses a 2 males × 2 females mating scheme, whereby two size-matched females and two randomly selected males are housed in the same enclosure. Males compete over access to females. Individual age was recorded as the number of years from birth to the time of our data collection based on captive records.

We retrieved the genetic relationships for each individual from the breeding centre’s studbook. Lizards were monitored daily in the breeding centre to ensure accurate allocation of maternity. We also established paternity using DNA. Buccal swabs were collected from all adults and neonates, and DNA was extracted using a Universal Genomic DNA Kit (CoWin Biosciences, Taizhou, China), according to a modified protocol (of E.Z.N.A.^®^ Forensic DNA Kit for buccal swabs). SNP genotyping was performed by Shanghai BioWing Applied Biotechnology Company using multiplex PCR with next-generation sequencing on the high-throughput genotyping platform, Illumina X-10 [29]. We conducted the paternity analysis with data from 98 SNPs using CERVUS 3.0.7.0 [30].

We also obtained data on reproductive output for each male and female, including litter size (total number of offspring sired, including stillborns), litter mass (total mass of offspring sired, including stillborns), total live number (total number of offspring sired, excluding stillborns) and total live mass (total mass of offspring sired, excluding stillborns). When there was mixed paternity, we summed up the mass of babies sired by the males of interest. We distinguished live from stillborn offspring because of the obvious fitness consequences and because females in captivity give birth to a large percentage of stillborns (16.3%, data obtained from the breeding centre).

### 2.7. Relationship between Colour, Morphology and Functional Performance Traits

We used LMM in R package *lme4* [26] to determine the relationship between colour and individual quality. For each lizard, we scored colour variant (red vs. non-red) and used SMI, bite force, head size, tail size and age as measures of individual quality. SMI was log-transformed and treated as a dependent variable, with colour variant as the independent variable, while assuming a Gaussian error distribution. To account for the effect of genetic relationships on body condition variation, we added a family group parameter, which describes the full sibling relationship between individuals as a random effect. The normality of model residuals was tested using “*qqnorm*” function in the R package *stats* and used as a measure of model goodness of fit [19]. We performed the same analysis for bite force, head size, tail size and individual age. For bite force, we added body temperature of the lizard as a covariate to account for the influence of body temperature on bite force measurement. We further examined the relationship between conspicuousness (JNDs of chromatic and luminance contrast) and the corresponding phenotypes for colour patches of the throat and venter separately.

### 2.8. Colour Variation and Reproductive Output

We examined the relationship between colour variant and reproductive output using a LMM in R. We used four measures of reproductive output: litter size, litter mass, total live mass and total live number. These four measures of reproductive output were dependent variables, while colour variants according to body regions were considered independent variables; we assumed a Gaussian error distribution. SVL was added as a covariate to account for body size on reproductive output. To account for genetic effects, we added a family group parameter, where full siblings were placed in a group, as a random effect. We performed this analysis separately for males and females. We further examined the relationship between conspicuousness (JNDs of chromatic and luminance contrast) and corresponding reproductive outputs for throat and venter colour (separately).

## 3. Results

### 3.1. Colour Variation

**Figure 2:**
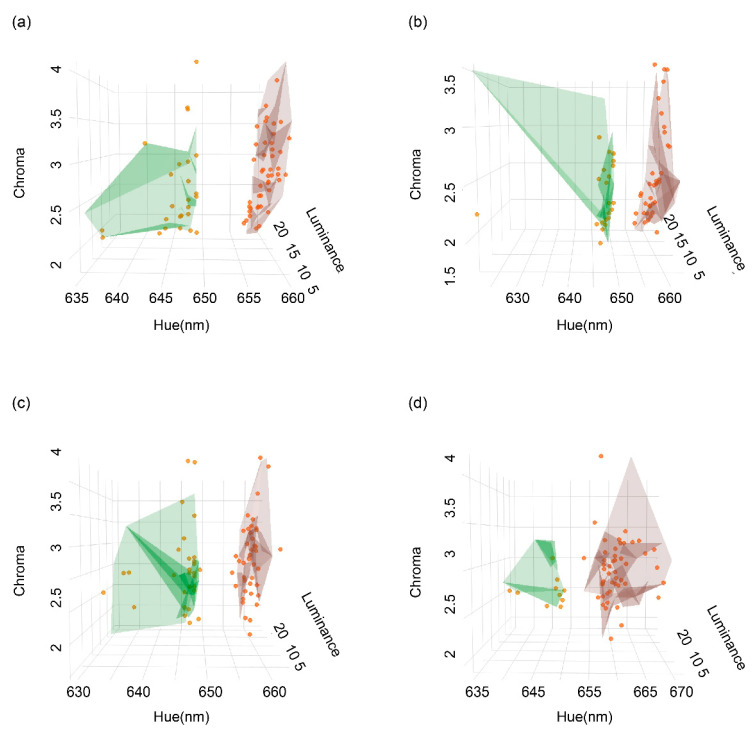
Results of a cluster analysis of hue, chroma and luminance by sex and body region (throat, venter). (**a**) Throat in females, (**b**) throat in males, (**c**) venter in females and (**d**) venter in males.

Based on spectral data (hue, chroma and luminance), individuals were classified as either red or non-red for the throat and venter for males and females, respectively (Figure 2, Table S1, Figure S1). Eighty-one individuals had red throats (36 males, 45 females), 46 individuals lacked red on their throat (21 males, 25 females), while 88 individuals had a red venter (47 males, 41 females) and 39 individuals lacked any red on their venter (10 males, 29 females). Throat colour was not correlated with ventral colour: 23 individuals had a red venter but a throat without any red, while 16 individuals had a red throat but a venter without red. However, the allocation of morphs based on spectral clustering did not always align with human perception based on their photos. Forty-three out of 69 males (62.32%) and 46 out of 58 females (79.31%) were classified as the same ventral colour morph, while 49 out of 69 males (71.01%) and 45 out of 58 females (77.59%) were classified as the same throat colour morph.

**Figure 3:**
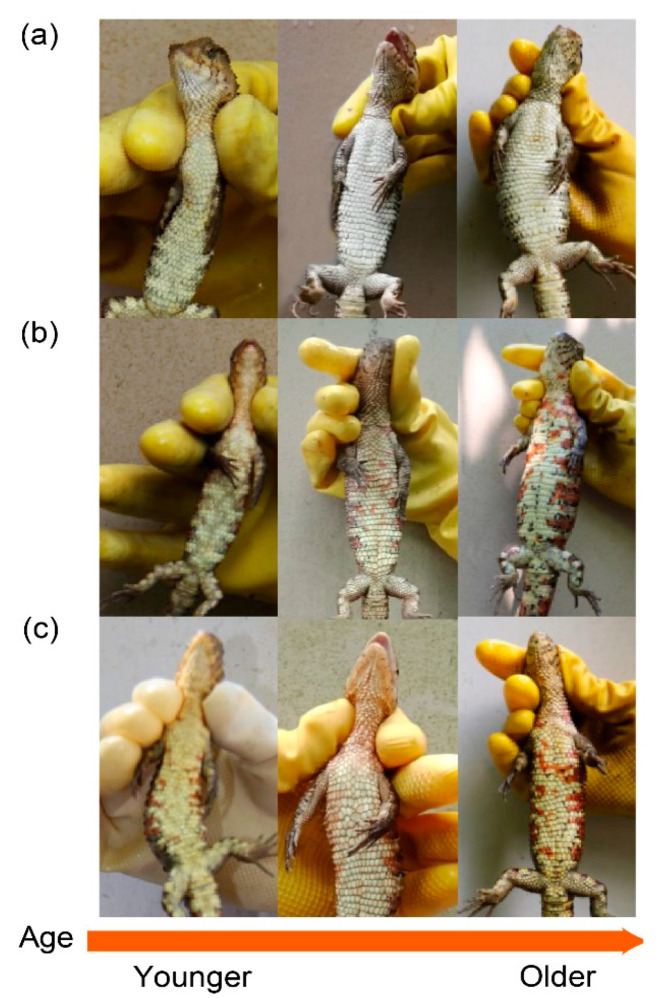
Ontogeny of colouration. (**a**) An individual born without red and which never developed red as it got older; (**b**) an individual born without red, but later developed red with age; (**c**) an individual born with red which became redder as it got older. The arrow at the bottom shows the direction of age change.

Some individuals appeared to change their colour types during development, while others did not. Fourteen individuals (six males and eight females) were born without red and never developed any red colouration as they aged. Three individuals (two males and one female) were born with red colour and retained red as they aged. Fifteen individuals (three males and twelve females) were born without red but developed red with age (Figure 3). Importantly, individuals that had red spots at birth or which developed red spots/blotches later, became increasingly red with age, which suggests that colour is a dynamic signal whose information content changes with age and/or condition.

### 3.2. Conspicuousness of Colour Patch to a Lizard Receiver

**Figure 4:**
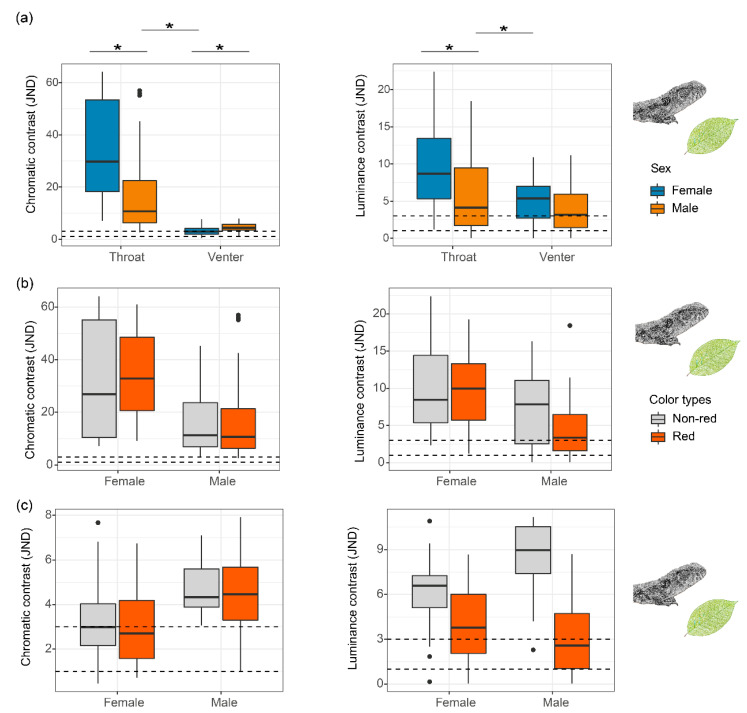
Conspicuousness of *S*. *crocodilurus*’ colour in just-noticeable differences (JNDs) in relation to a lizard visual system for chromatic and luminance channels against a green leaf background. (**a**) Chromatic and luminance contrast of colour patch by sex and body region. (**b**) Chromatic and luminance contrast of throat colour patch by colour variant (red, no-red) and sex. (**c**) Chromatic and luminance contrast of ventral colour patch by colour variant (red, no-red) and sex. “*” indicates a significant difference. Black points in the figure show the outliers of conspicuousness.

Average JND values of chromatic and luminance contrast against background vegetation were greater than three for both the throat and the venter for both sexes, suggesting their colours are easily discriminable to a lizard receiver and likely to be important for social communication (Table 1, Figure 4a, Table S2). Throat chromatic and luminance contrast in both sexes were significantly greater than those of the venter (Table 1, Figure 4a, Table S2), suggesting that the throat was more conspicuous and possibly more important for signalling than the venter. Surprisingly, female throat chromatic and luminance contrast was significantly greater than males, while the chromatic contrast of the venter was significantly greater in males than females (Table 1, Figure 4a, Table S2).

When we separately analysed red and non-red colour variants, chromatic and luminance contrast against vegetation backgrounds were greater than three in both sexes and body regions (Table 1, Figure 4b,c), confirming that red and non-red colour variants can be easily discriminated by lizards. We did not detect a significant difference in conspicuousness between red and non-red colour variants for throat colour for both sexes, but the luminance contrast of the venter was significantly greater in non-red than in red individuals, for both males and females (Table 1, Figure 4b,c, Table S3).

### 3.3. Relationships between Colour Variation and Phenotypic Traits

**Figure 5:**
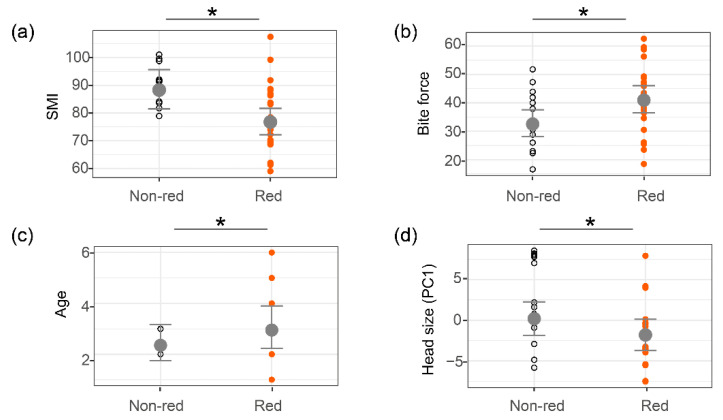
Relationship between (**a**) throat colour variants (non-red, red) and scaled mass index (SMI) of females; (**b**) throat colour variants and bite force in females; (**c**) throat colour variants and age in males; (**d**) ventral colour variants and head size in females. Orange points show the trait value of red variant, and black points show the trait value of non-red variant. Grey points are the predicted average value, and error bars represent the 95% confidence interval. “*” indicates significant association. See Table 2 for nonsignificant relationships.

**Figure 6:**
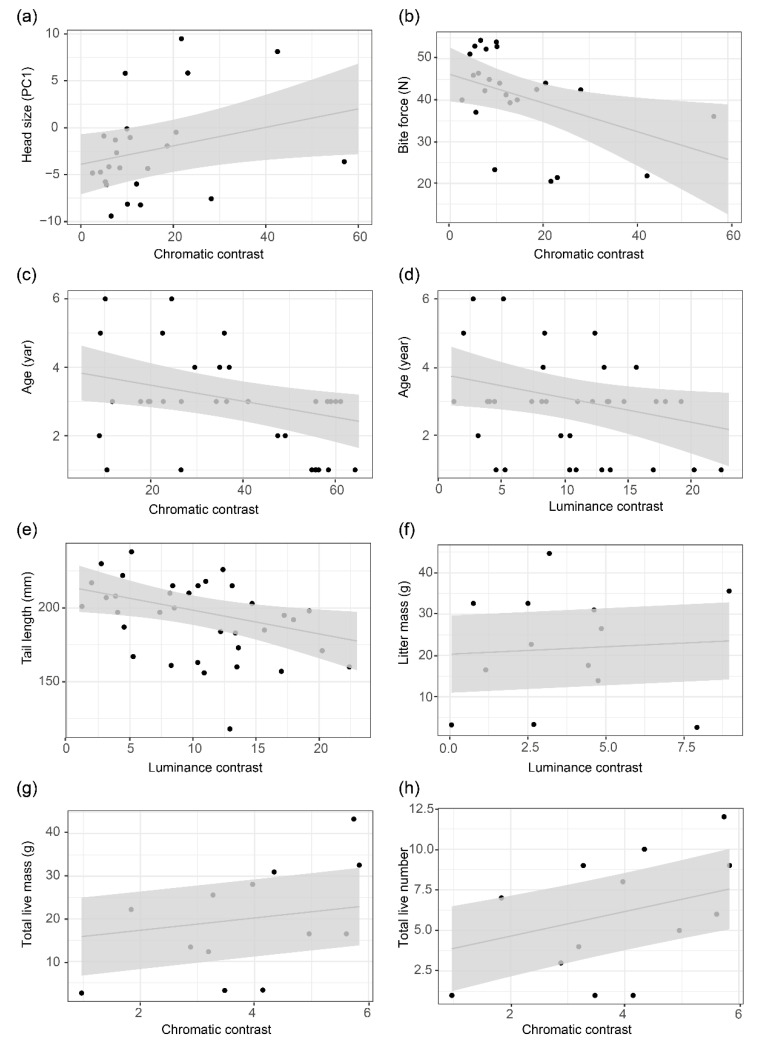
Relationship between phenotypic traits, reproductive output, and conspicuousness of colour patch in just-noticeable differences (JNDs) in relation to a lizard visual system for chromatic and luminance channels against a common vegetation background. (**a**) Chromatic contrast in throat colour of males in relation to head size (PC1); (**b**) chromatic contrast in throat colour of males in relation to bite force; (**c**) chromatic contrast in throat colour of females in relation to individual age; (**d**) luminance contrast in throat colour of females in relation to individual age; (**e**) tail length and luminance contrast in throat colour of females; (**f**) luminance contrast in ventral colour of males in relation to the litter mass of the offspring they sired; (**g**) chromatic contrast in ventral colour of males in relation to the total live mass of the offspring they sired; (**h**) chromatic contrast in ventral colour of males in relation to total live number of the offspring they sired. Grey line means the predicted phenotypic value against conspicuousness of colour patch based on linear mixed model and grey area represents the 95% confidence intervals.

All relationships between colour variants and phenotypic traits are depicted or summarised in Figure 5 and Figure 6, Table 2, Table 3, and Table S4. In males, throat colour was positively and significantly related to age (estimate = 0.23, t = 2.60, *p* = 0.03); individuals with a red throat were older (3.12 ± 1.62 years) than those with a non-red throat (2.86 ± 0.38 years, Figure 5c, Table 2, Table S4). Further, we found a positive and significant relationship between head size (PC1) and chromatic contrast in throat (estimate = 0.10, t = 2.08, *p* = 0.05, Figure 6a, Table 3), while bite force was negatively and significantly correlated with chromatic contrast in throat (estimate = −0.01, t = −2.32, *p* = 0.03, Figure 6b, Table 3). Males with colourful (greater chromatic contrast) throats had smaller heads and weaker bite force. No relationship was found between ventral colour variation (colour variant, conspicuousness) and body condition, bite force, morphology and age in males.

In females, throat colour was significantly related to individual SMI (estimate = −0.14, t = −2.95, *p* = 0.01) and bite force (estimate = 0.23, t = 3.09, *p* = 0.01). Females with a red throat (SMI = 78.56 ± 12.33) had a slender body shape compared to those with a non-red throat (SMI = 90.25 ± 7.20, Figure 5a, Table 2, Table S4). Females with a red throat had greater bite force (42.31 ± 12.32 N) than those with a non-red throat (34.18 ± 11.15 N, Figure 5b, Table 2, Table S4). Furthermore, ventral colour was significantly related to head size (estimate = −2.00, t = −2.22, *p* = 0.04); females with a red venter had larger heads than those with non-red venters (Figure 5d, Table 2, Table S4). We found a negative and significant relationship between age and chromatic contrast (estimate = −0.01, t = −2.41, *p* = 0.03, Figure 6c, Table S5) and between age and luminance contrast (estimate = −0.02, t = −2.02, *p* = 0.05, Figure 6d, Table 4) in throat colour in females. Tail length was negatively and significantly correlated with luminance contrast in throat. Younger females had shorter tails and were more conspicuous (chromatic and luminance contrast) in throats (estimate = −0.01, t = − 2.37, *p* = 0.02, Figure 6e, Table 3). No relationship was found between conspicuousness of ventral colour and morphology, age, body condition and bite force in females.

### 3.4. Relationship between Colour Variation and Reproductive Output

All relationships between colour variation and reproductive output are depicted or summarised in Figure 6, Table 3 and Table 4 and Table S5. Among the four reproductive output parameters that we examined, no significant difference was detected between red and non-red colour variants by body region and sex (Table 4, Table S5). We found a positive and significant relationship between litter mass and luminance contrast in male ventral colour (estimate = 0.01, t = 6.63, *p* = 0.02, Figure 6f, Table 3). We also found a positive and significant relationship between the total mass of live offspring sired by males and ventral chromatic contrast (estimate = 0.08, t = 23.71, *p* < 0.01, Figure 6g, Table 3) and between the total number of live offspring sired by a male and ventral chromatic contrast of males. Males with greater ventral luminance contrast had larger offspring, and individuals with a more colourful (greater chromatic contrast) venter sired more live offspring overall, and these offspring were larger (estimate = 0.16, t = 3.02, *p* = 0.04, Figure 6h, Table 3).

## 4. Discussion

Our research quantified throat and ventral colour variation in male and female *S. crocodilurus* and examined its potential relationship with individual quality. Males and females were sexually dimorphic in colouration although both sexes had a red morph. Females had more conspicuous throats (both luminance and chromatic contrast) than males while males had more conspicuous venters (chromatic contrast) than females, suggesting different roles in signalling and possibly different selective pressures on males and females. Males and females clustered into the discrete throat and ventral morphs using traditional spectral measures of hue, luminance and chroma although throat and ventral colour were uncoupled. We also examined changes in colour over time using photographs and found that colour (particularly red) changed with age. Male and female colouration was easily discriminable, suggesting a social role for colour. Furthermore, throat colour, and to a lesser extent ventral colour, was linked to morphology, life history and performance traits in different ways in males and females, suggesting that colour signals have the potential to convey key information about individual quality.

A major aim of this study was to determine whether crocodile lizards are polymorphic for colour such that colour patches are discrete. We used spectral measures of hue, chroma and luminance of the throat and venter, separately, to determine if lizards could be allocated to morphs. Males and females could reliably be allocated to either a throat or ventral morph using cluster analysis, but this did not always align with human perception based on their photos. We found no link between the throat and ventral colour which suggests that if they have a signalling function, they likely convey different information. We were also able to make use of a photographic database of the captive colony for a limited number of individuals that were tracked from their first year. We were able to subjectively determine that as lizards with red pigment aged, they accumulated pigment. Throat colour was therefore not static but changed with age, which is consistent with a signal of individual quality. Old individuals often have more breeding and foraging experience and have improved survival and reproductive performance [31]. At this stage, it is premature to suggest that crocodile lizards have discrete colour polymorphisms because our study represents a single snapshot in time where the population was objectively measured for colour only once. The intensity of colouration likely changes with the season and depends on the time of shedding. Additionally, with some exceptions, most lizard systems with discrete colour morphs can readily be identified by a human observer [2,10].

Crocodile lizards have substantial colour variation that not only varies between sexes but also within sexes. We focused on two body regions, the throat and venter, and examined their relationship to a range of traits. The ventral colour was unrelated to morphology, age, body condition and bite force in males. Interestingly, the throat was more conspicuous than the venter. In lizards more broadly, throat colour is a more common signal in social interactions [32], and therefore, this may have favoured selection on conspicuousness of the throat over the venter. Throat colour was related to body condition in females, bite force in females and age in males, suggesting the possibility that throat colour might signal social dominance and/or fighting ability. Males in particular will engage in intense contests with lots of biting and perform head-bob displays to show their throat colour to rivals [33]. Staged contests are required to elucidate the potential role of colour as a status signal. The fact that throat and ventral colour are uncoupled and signal different information, while also corresponding to specific morphs, suggests a complex system that could be under both natural and sexual selection in different ways in males and females. Similar results have been found in other species. For example, in male tree lizards (*Urosaurus ornatus*) the belly and throat patches are displayed simultaneously during territorial encounters and signal different information. Dominant male *U. ornatus* with small belly patches have relatively low bite force while subordinates may have large belly patches and high bite force [34]. Likewise, in *Sceloporus consobrinus*, abdominal and throat hue do not covary and likely have independent functions [35].

Red and yellow pigmentation occurs in numerous lizard species and may be due to carotenoids (acquired through diet), pteridines (self-synthesised), or both [36,37]. Red pigments are thought to be an honest signal of quality because carotenoids can be rare in the environment or the production costs involved in, for example converting yellow carotenoids to red ketocarotenoids, are high [37]. We did not determine the specific red pigments in crocodile lizards; however, on the assumption that they represent a production cost, it is informative to examine their relationship with a range of traits that bear on fitness. In males, a key finding was that red males lived longer than males without red. This result was slightly surprising because males that invest in signal production are thought to be trading off against immune function or other physiological processes. In females, individuals with red on their throats had greater bite force than individuals lacking red on their throats, and they tended to be more slender (lower SMI). In males, bite force was lower in males with redder throats (higher chromatic contrast). This is similar to the finding in wall lizards, *P. muralis*, that individuals with more red had lower sprint speed compared to individuals with less red [38], suggesting a trade-off between physiological performance and red pigmentation.

We also examined direct links between colour and fitness (reproductive output). Although life history strategy (e.g., r- vs. K-strategist) has been documented in the females of multiple colour polymorphic species (reviewed in [36]), this was not the case here. We found no link between any measure of reproductive output and colour in females. We did, however, find links between male life history traits and colour. Most significantly, males with brighter venters (greater luminance contrast) sired larger offspring. Males with greater ventral chromatic contrast also sired more and larger live offspring overall. Furthermore, red males tended to live longer. This raises the intriguing possibility of discrete life history strategies associated with colour, at least in males. Studies in the wild and incorporating a wider range of life history traits would be beneficial for determining this.

Given that crocodile lizards are an endangered species, it is worth considering the conservation implications of these findings. Preserving diversity is an overarching goal of conservation biology. Given the range of variation in colour and the different links between males and females with respect to morphology, performance and life history, it is important to capture this diversity in translocation or release projects. This diversity may facilitate local adaptation during environmental fluctuations. We encourage future captive breeding and release programs to incorporate colour diversity within and between the sexes and to track individuals released back into the wild.

## 5. Conclusions

Different colour morphs were detected for the throat and venter, but it is premature to claim that crocodile lizards have discrete colour morphs because colour variation in throat and venter is not correlated. Colour variation in the throat and venter is thought to function in social communication and signal individual quality, but the information signalled is likely to be different between sexes and body regions. We also found some links between colour variation and reproductive output in the venter, but only in males. The relationship between colour variation and individual quality could be useful in captive breeding and release programs for this endangered species. Future research should focus on examining potential links between colour variation and social behaviour, including alternate reproductive tactics.

## Figures and Tables

**Table 1:** Comparisons of means and standard deviations for chromatic and luminance contrast (JNDs) for red and non-red colour variants against background vegetation under a lizard visual system for each sex. Bold indicates significant difference (*p* < 0.05) between two colour variants.

JNDs	Female (n = 70)		Male (n = 57)	
Throat (n = 127)	Non-red (n = 25)	Red (n = 45)	Non-red (n = 21)	Red (n = 36)
Chromatic	30.77 ± 20.20	36.34 ± 17.05	15.02 ± 11.90	20.41 ± 17.88
Luminance	9.62 ± 5.75	10.23 ± 5.25	7.04 ± 5.17	6.37 ± 5.75
Venter (n = 127)	Non-red (n = 29)	Red (n = 41)	Non-red (n = 10)	Red (n = 47)
Chromatic	3.29 ± 1.71	3.21 ± 1.55	4.53 ± 1.31	4.62 ± 1.74
Luminance	**6.40 ± 2.47**	**3.87 ± 2.33**	**7.55 ± 3.32**	**3.65 ± 3.10**

**Table 2:** Summary of means and standard deviations of phenotypic traits for two colour variants (non-red, red) for each body region and sex. PC1 of head size explained 86% of the total variation in head length, width and height, and PC1 was negatively correlated with head length, width and height. Bold indicates significant difference (*p* < 0.05) between two colour variants.

		Throat (n = 59)		Venter (n = 59)	
Female (n = 35)	Combined (n = 35)	Non-Red (n = 12)	Red (n = 23)	Non-Red (n = 16)	Red (n =19)
Age (year)	2.83 ± 1.44	2.75 ± 1.71	2.87 ± 1.32	2.50 ± 1.32	3.11 ± 1.52
SMI	82.57 ± 12.12	**90.25 ± 7.20**	**78.56 ± 12.33**	85.29 ± 9.62	80.28 ± 13.71
Head size (PC1)	0.07 ± 4.86	0.89 ± 5.49	−0.36 ± 4.56	**2.40 ± 4.90**	**−1.89 ± 3.96**
Head length (mm)	29.63 ± 3.21	29.51 ± 3.79	29.69 ± 2.96	**28.03 ± 3.22**	**30.97 ± 2.58**
Head width (mm)	23.62 ± 2.82	23.07 ± 3.23	23.90 ± 2.62	**22.28 ± 2.92**	**24.74 ± 2.24**
Head height (mm)	18.30 ± 3.67	17.38 ± 2.59	18.79 ± 4.09	**17.26 ± 3.85**	**19.18 ± 3.36**
Tail length (mm)	192.54 ± 26.28	184.33 ± 31.76	196.83 ± 22.52	182.19 ± 19.96	201.26 ± 28.22
Bite force (n)	39.52 ± 12.40	**34.18 ± 11.15**	**42.31 ± 12.32**	36.61 ± 13.28	41.97 ± 11.39
**Male (n = 24)**	**Combined (n = 24)**	**Non-red (n = 7)**	**Red (n = 17)**	**Non-red (n = 5)**	**Red (n = 19)**
Age (year)	3.04 ± 1.37	**2.86 ± 0.38**	**3.12 ± 1.62**	3.40 ± 0.55	2.95 ± 1.51
SMI	81.47 ± 7.81	80.62 ± 12.77	81.83 ± 5.11	82.65 ± 10.30	81.17 ± 7.34
Head size (PC1)	−2.35 ± 5.13	−3.06 ± 1.87	−2.06 ± 6.01	−3.43 ± 1.42	−2.06 ± 5.72
Head length (mm)	31.13 ± 3.83	31.86 ± 1.31	30.84 ± 4.49	32.21 ± 0.67	30.85 ± 4.27
Head width (mm)	25.54 ± 2.92	25.87 ± 1.86	25.40 ± 3.29	25.66 ± 1.50	25.50 ± 3.22
Head height (mm)	19.01 ± 2.55	19.07 ± 1.08	18.99 ± 2.98	19.57 ± 0.64	18.86 ± 2.84
Tail length (mm)	202.21 ± 20.23	209.00 ± 16.05	199.41 ± 21.52	209.80 ± 13.76	200.21 ± 21.46
Bite force (n)	41.33 ± 10.40	44.23 ± 4.10	40.14 ± 12.00	47.44 ± 4.19	39.72 ± 11.01

**Table 3:** Summary of a linear mixed model (LMM) examining correlation between colour conspicuousness in just-noticeable differences (JNDs) in relation to a lizard visual system for chromatic and luminance channels against background vegetation, individual quality-related traits and reproductive output by body region and sex. Bold indicates a significant association (*p* < 0.05).

		Female (N = 16)			Male (N = 13)		
		Estimate	t Value	*p* Value	Estimate	t Value	*p* Value
Throat							
Chromatic contrast	Age	**−0.01**	**−2.41**	**0.03**	−0.01	−1.73	0.11
	SMI	−0.002	−1.14	0.27	0.001	0.31	0.76
	Head size (PC1)	0.01	0.50	0.62	**0.10**	**2.08**	**0.05**
	Tail length	−0.002	−1.77	0.09	−0.002	−1.45	0.16
	Bite force	−0.003	−1.27	0.21	**−0.01**	**−2.32**	**0.03**
	Litter size	0.001	0.17	0.86	0.01	0.54	0.60
	Litter mass	0.01	1.19	0.26	0.001	0.06	0.96
	Total live mass	0.02	1.54	0.15	−0.02	−1.40	0.25
	Total live number	0.01	1.48	0.16	0.01	0.28	0.79
Luminance contrast	Age	**−0.02**	**−2.02**	**0.05**	−0.02	−1.82	0.10
	SMI	−0.005	−0.95	0.35	0.001	0.25	0.81
	Head size (PC1)	0.10	1.03	0.31	0.13	1.65	0.13
	Tail length	**−0.01**	**−2.37**	**0.02**	−0.002	−1.03	0.33
	Bite force	−0.01	−1.34	0.19	−0.01	−1.03	0.32
	Litter size	−0.01	−0.95	0.36	−0.04	−0.90	0.39
	Litter mass	0.001	0.05	0.96	−0.02	−1.06	0.35
	Total live mass	0.03	0.84	0.42	0.04	1.00	0.34
	Total live number	0.03	0.87	0.40	0.02	0.50	0.63
Venter							
Chromatic contrast	Age	−0.05	−1.45	0.16	−0.03	-0.95	0.37
	SMI	−0.01	−0.58	0.57	−0.02	−1.05	0.31
	Head size (PC1)	0.42	1.23	0.23	0.21	0.64	0.54
	Tail length	0.0	−0.21	0.83	−0.01	−0.66	0.52
	Bite force	0.01	0.28	0.78	−0.04	−1.24	0.24
	Litter size	−0.06	−1.32	0.21	0.03	0.45	0.68
	Litter mass	−0.02	−0.30	0.77	−0.01	−0.58	0.61
	Total live mass	0.11	0.82	0.43	**0.08**	**23.71**	**0.002**
	Total live number	0.08	0.71	0.49	**0.16**	**3.02**	**0.04**
Luminance contrast	Age	−0.02	−1.19	0.25	0.002	0.15	0.88
	SMI	0.003	0.28	0.78	−0.003	−0.46	0.65
	Head size (PC1)	0.07	0.41	0.69	0.09	0.65	0.53
	Tail length	0.004	0.60	0.56	−0.001	−0.36	0.73
	Bite force	−0.002	−0.15	0.88	0.02	1.36	0.19
	Litter size	0.02	1.05	0.31	0.01	0.43	0.70
	Litter mass	−0.02	−0.63	0.54	**0.01**	**6.63**	**0.02**
	Total live mass	−0.09	−1.60	0.14	−0.01	−0.55	0.64
	Total live number	−0.06	−1.09	0.29	−0.03	−0.74	0.51

**Table 4:** Summary of means and standard deviations of reproductive output (offspring) traits for two colour variants (non-red, red) of each body region and sex. No significant difference was found between two colour variants in all cases.

		Throat (n = 29)		Venter (n = 29)	
Female (n = 16)	Combined (n = 16)	Non-red (n = 7)	Red (n = 9)	Non-red (n = 10)	Red (n = 6)
Litter size	7.31 ± 2.18	7.57 ± 0.98	7.11 ± 2.85	6.80 ± 1.32	8.17 ± 3.13
Litter mass	21.60 ± 7.29	21.91 ± 6.91	21.36 ± 7.98	19.60 ± 6.08	24.93 ± 8.47
Total live mass	20.04 ± 8.68	20.37 ± 9.72	19.78 ± 8.38	18.14 ± 8.04	23.20 ± 9.51
Total live number	6.31 ± 2.91	6.43 ± 2.70	6.22 ± 3.23	5.50 ± 2.32	7.67 ± 3.50
**Male (n = 13)**	**Combined (n = 13)**	**Non-red** **(** **n = 5** **)**	**Red (n = 8)**	**Non-red** **(** **n = 1** **)**	**Red (n = 12)**
Litter size	7.46 ± 4.96	7.20 ± 4.82	7.63 ± 5.37	14.00	6.92 ± 4.76
Litter mass	21.75 ± 13.60	21.48 ± 13.59	21.93 ± 14.55	35.60	20.60 ± 13.53
Total live mass	19.28 ± 12.63	18.42 ± 12.11	19.83 ± 13.74	28.10	18.55 ± 12.90
Total live number	5.85 ± 3.69	5.40 ± 3.21	6.13 ± 4.16	8.00	5.67 ± 3.80

## Data Availability

The data presented in this study are openly available in ScienceDB (Science data bank) at doi: 10.57760/sciencedb.02354, reference number 31253.11.sciencedb.02354.38.

## Supplementary Materials

The following supporting information can be downloaded at: https://www.mdpi.com/article/10.3390/biology11091314/s1, Figure S1: Best k value for clustering analysis using sum of squares (SSE) optimized by the elbow method; Table S1: Summary of means and standard deviations of the color traits for two color variants (non-red, red) of each body region and each sex; Table S2: Summary of a linear mixed models (LMM) examining differences of color conspicuousness in Just Noticeable Differences (JNDs) in relation to a lizard visual system for chromatic and luminance channels against background vegetation between sexes and body regions, and interaction between them. F: female, M: male, T: throat, V: venter. Bold indicates a significant difference (*p* < 0.05); Table S3: Summary of a linear mixed models (LMM) examining differences of color conspicuousness in Just Noticeable Differences (JNDs) in relation to a lizard visual system for chromatic and luminance channels against background vegetation between two color variants. Summary of a linear mixed models (LMM); Table S4: Summary of a linear mixed models (LMM) examining differences of individual quality related traits between two color variants (red, non-red) by body region and sex. Summary of a linear mixed models (LMM); Table S5: Summary of a linear mixed models (LMM) examining differences of reproductive output traits between two color variants (non-red, red) by body region and sex.
