# Supplementary material for: Colour Variation in the Crocodile Lizard (Shinisaurus crocodilurus) and Its Relationship to Individual Quality
Source: Biology (Basel). 2022 Sep 4;11(9):1314. doi: 10.3390/biology11091314 (PMC9495974; doi:10.3390/biology11091314)
Supplement: Supplementary file 1 [file biology-11-01314-s001.zip › biology-1890652-supplementary.pdf]

Supplementary Figures and Tables

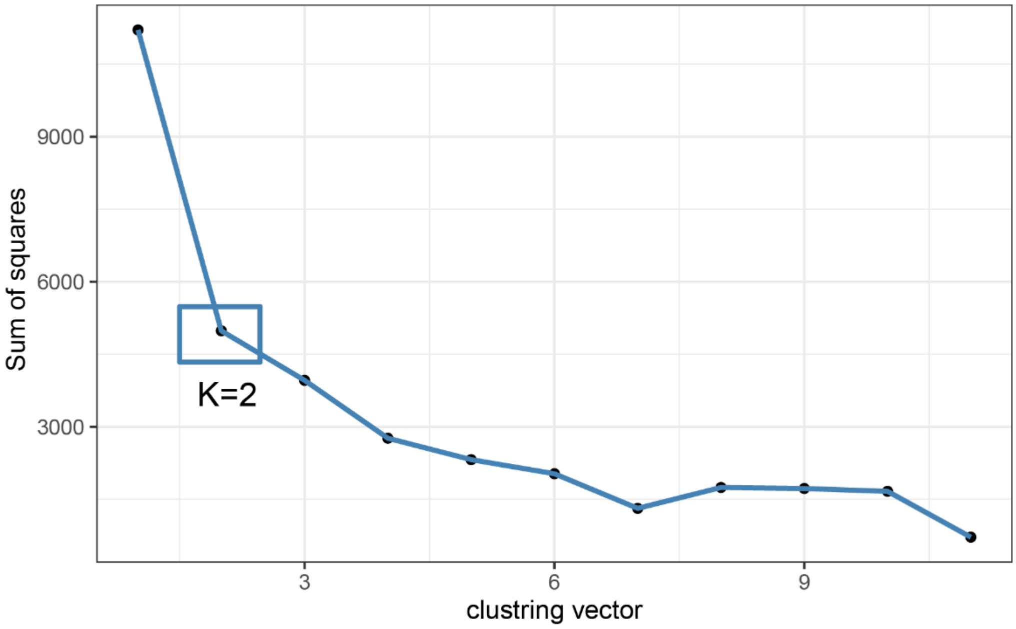

Figure S1. Best k value for clustering analysis using sum of squares (SSE) optimized by the elbow method.

Table S1. Summary of means and standard deviations of the color traits for two color variants (non-red, red) of each body region and each sex.

	Throat (n=127)			Venter (n=127)		
Female (n=70)	Combined (n=70)	Non-red (n=25)	Red (n=45)	Combined (n=70)	Non-red (n=29)	Red (n=41)
Hue	653.41 ± 5.95	646.16 ± 3.77	657.44 ± 1.10	652.90 ± 7.29	645.24 ± 4.98	658.32 ± 1.31
Chroma	2.78 ± 0.50	7.04 ± 5.80	4.96 ± 2.43	1.44 ± 0.30	18.45 ± 5.66	15.35 ± 4.13
Luminance	5.70 ± 4.06	2.61 ± 0.59	2.88 ± 0.43	16.63 ± 5.03	1.39 ± 0.31	1.49 ± 0.28
Male (n=57)	Combined (n=57)	Non-red (n=21)	Red (n=36)	Combined (n=57)	Non-red (n=10)	Red (n=47)
Hue	654.25 ± 6.86	646.71 ± 5.77	658.64 ± 1.55	658 ± 7.12	645.20 ± 5.07	660.72 ± 3.67
Chroma	2.40 ± 0.47	7.08 ± 4.37	8.50 ± 4.59	1.84 ± 0.41	21.67 ± 6.88	13.59 ± 4.83
Luminance	7.98 ± 4.52	2.29 ± 0.37	2.47 ± 0.52	15.01 ± 6.03	1.54 ± 0.25	1.90 ± 0.41

Table S2. Summary of a linear mixed models (LMM) examining differences of color conspicuousness in Just Noticeable Differences (JNDs) in relation to a lizard visual system for chromatic and luminance channels against background vegetation between sexes and body regions, and interaction between them. F: female, M: male, T: throat, V: venter. Bold indicates a significant difference (*P* < 0.05).

JNDs	Fixed effect	Estimate	t value	*P* value		Mean ± SD	
						Throat	Venter
Chromatic	F vs M	-14.99	-6.32	**<0.001**	F	34.76 ± 18.16	3.18 ± 1.62
	T vs V	-30.16	-14.40	**<0.001**	M	18.60 ± 16.26	4.47 ± 1.66
	Sex : region	17.46	5.55	**<0.001**			
Luminance	F vs M	-3.82	-4.51	**<0.001**	F	10.99 ± 5.85	4.70 ± 2.70
	T vs V	-4.94	-6.48	**<0.001**	M	7.16 ± 5.95	4.33 ± 3.41
	Sex : region	3.18	2.78	**<0.001**			

Table S3. Summary of a linear mixed models (LMM) examining differences of color conspicuousness in Just Noticeable Differences (JNDs) in relation to a lizard visual system for chromatic and luminance channels against background vegetation between two color variants. Summary of a linear mixed models (LMM).

JNDs	Female (non-red vs red)			Male (non-red vs red)		
Throat (n=97)	Estimate	t value	*P* value	Estimate	t value	*P* value
Chromatic	0.23	1.39	0.17	-0.28	-1.29	0.20
Luminance	0.04	0.22	0.82	-0.44	-1.08	0.29
Venter (n=97)						
Chromatic	-0.11	-0.66	0.51	-0.15	-1.09	0.28
Luminance	**-0.57**	**-2.12**	**0.04**	**-1.32**	**-3.45**	**0.001**

Table S4. Summary of a linear mixed models (LMM) examining differences of individual quality related traits between two color variants (red, non-red) by body region and sex. Summary of a linear mixed models (LMM).

	Female (non-red vs red)			Male (non-red vs red)		
	Estimate	t value	*P* value	Estimate	t value	*P* value
Throat						
**Age**	-0.10	-0.98	0.34	**0.23**	**2.60**	**0.03**
**SMI**	**-0.14**	**-2.95**	**0.01**	0.02	0.53	0.60
Head size (PC1)	0.08	0.08	0.94	-1.88	-2.12	0.05
Tail length	0.08	1.87	0.07	0.004	0.15	0.89
**Bite force**	**0.23**	**3.09**	**0.01**	-0.12	-1.11	0.28
Venter						
Age	0.05	0.46	0.65	-0.02	-0.20	0.84
SMI	-0.04	-0.86	0.40	-0.02	-0.31	0.76
**Head size (PC1)**	**-2.00**	**-2.22**	**0.04**	-0.59	-0.59	0.57
Tail length	0.05	1.13	0.27	0.004	0.06	0.95
Bite force	0.13	1.63	0.11	-0.18	-1.87	0.09

Table S5. Summary of a linear mixed models (LMM) examining differences of reproductive output traits between two color variants (non-red, red) by body region and sex.

	Female (non-red vs red)			Male (non-red vs red)		
	Estimate	t value	*P* value	Estimate	t value	*P* value
Throat						
Litter size	-0.03	-0.26	0.80	0.19	0.69	0.52
Litter mass	0.07	0.49	0.63	-0.17	-1.53	0.19
Total live mass	0.27	0.87	0.40	-0.21	-1.13	0.29
Total live number	0.19	0.67	0.52	-0.04	-0.14	0.90
Venter						
Litter size	0.03	0.22	0.83	-	-	-
Litter mass	0.14	1.00	0.34	-	-	-
Total live mass	0.22	0.66	0.52	-	-	-
Total live number	0.20	0.69	0.50	-	-	-
